# Liposomal J-Aggregates of Indocyanine Green as a Multifunctional Contrast Agent for Photoacoustic Imaging and Photothermal Therapy

**DOI:** 10.7150/ntno.123184

**Published:** 2026-02-11

**Authors:** Noah Stern, Binita Shrestha, Susan Burrell, James Tunnell, Tyrone Porter

**Affiliations:** Biomedical Engineering Department, University of Texas at Austin, Austin, TX, USA.

**Keywords:** indocyanine green, J-aggregates, photoacoustic imaging, photothermal therapy, theranostic

## Abstract

One of the major barriers to further clinical adoption of photoacoustic imaging (PAI) and photothermal therapy (PTT) is a lack of versatile and multi-faceted contrast agents. While indocyanine green (IcG) has gained considerable attention as a promising contrast agent, it is severely limited by rapid clearance time, low photostability, and a peak absorbance wavelength that coincides with hemoglobin. Herein, Liposomal J-aggregates of Indocyanine green (LJA) are presented as a superior alternative to generic IcG. They are a facilely produced, biodegradable nanoparticle offering advantageous physiochemical properties. J-aggregation results in a redshifted peak absorbance of increased magnitude and higher photostability compared to IcG. Liposome encapsulation increases circulation time leading to improved tumor uptake. Monte Carlo modeling of light interaction with tissue suggests that these improved optical properties make LJA a better contrast agent for PTT not only at longer wavelengths like 852nm and 890nm, but also at the commonly available and widely used 808nm. *In vitro* and *in vivo* testing support first approximations from modelling as LJA provide significantly higher photoacoustic signal, show increased tumor uptake, reach significantly higher temperatures under photothermal irradiation, and have the capacity to reduce tumor growth following therapy.

## 1. Introduction

Biomolecules and particles that absorb light in the near-infrared (NIR) region are extremely attractive clinically for various diagnostic and therapeutic applications. Photoacoustic imaging (PAI) is a versatile imaging modality that has gained considerable momentum in recent years for a variety of clinical uses including oncology.^1^-^6^ PAI offers real-time, high-resolution, functional and molecular imaging at depths of up to several centimeters in humans without the need for ionizing radiation. This is achieved through the detection of acoustic waves generated following the absorption of pulsed electromagnetic energy, a phenomenon called the photoacoustic effect.^7^ With the use of deeply penetrating first (NIR-I, 700-1000nm) and second (NIR-II, 1100 - 2000nm) near-infrared pulsed lasers and conventional ultrasound transducers, PAI combines the high resolution and specificity of optical imaging with the improved depth and usability of ultrasound. PAI exists chiefly as a diagnostic modality, however, when pulsed lasers are replaced with continuous lasers, laser irradiation leads to more sustained heat generation. Typically referred to as photothermal therapy (PTT), this technique can be used to locally heat tissue with a variety of therapeutic effects.^8^ It is well-known that the length and magnitude of heating can lead to immunogenic cell death, apoptosis, and even direct necrosis in irradiated cells.^9^-^13^ As such, local heating induced by PTT has become an attractive technique for cancer therapy either alone, as a co-therapy, or as a drug release modality.^14^-^19^

Both photoacoustic signal generation and photothermal heat generation are directly related to light absorption in tissue. Diagnostic imaging with PAI relies on known differences in optical absorption spectra between molecules to differentiate structures and the therapeutic use of PTT relies on absorbers to generate sufficient heat levels. At NIR wavelengths, key endogenous components include oxygenated hemoglobin, deoxygenated hemoglobin, water, melanin, and fat.^4^,^20^ Endogenous agents alone provide considerable clinical potential as seen with blood/tissue oxygenation estimation with PAI^21^-^23^ and direct laser-ablation with PTT^24^,^25^, but the introduction of effective exogenous agents could increase the potential uses and benefits of both PAI and PTT. As both PAI and PTT rely on optical absorbance, ideal contrast agents for PAI are often usable for PTT and vice versa. The ideal exogenous contrast agent for these methods would have high optical absorption, high photostability, be both biocompatible and biodegradable, and enable targeted imaging/therapy at greater depths in human tissue, however no single agent has emerged which satisfies each of these criteria.^26^-^28^ Limited availability of effective exogenous contrast agents remains a major barrier to further clinical adoption and expansion of PAI and PTT.^29^

Potential exogenous contrast agents include metallic nanoparticles, like gold^17^,^30^-^34^ and copper sulfide^35^,^36^, polymeric particles, like porphyrin-based^37^,^38^ and cellulose-based^39^,^40^, and small organic molecules like methylene blue^41^,^42^ and phthalocyanine^43^-^45^. One of the most promising exogenous agents for PAI and PTT is the small organic NIR dye, indocyanine green (IcG). The molecule has peak absorption between 790nm and 810nm and has been approved by the FDA for clinical use as an optical imaging contrast agent. While numerous studies have shown the promise of IcG for PAI and PTT applications^18^,^46^-^57^, it does have several shortcomings. IcG exhibits a short circulation half-life and is highly susceptible to photodegradation. Circulation time can be improved by encapsulation of IcG within a nanoparticle carrier.^47^,^48^,^50^-^52^ Encapsulation within a liposome offers considerable advantages. Liposome encapsulation of IcG has been shown to significantly prolong half-life without disruption of the optical properties that make it valuable for fluorescence imaging, photoacoustics, and phototherapy.^58^-^60^ In some instances, such as encapsulation with DOTAP, not only are circulation kinetics improved, but the binding of IcG to the lipids can also enhance its optical response by increasing stability and reducing degradation.^61^ As a vast majority of FDA-approved nanomaterials are either liposomes or lipid nanoparticles, robust combinations of lipids and cyanine dyes like IcG show considerable promise for contrast-assisted diagnostics and therapies.

Beyond advantageous combinations with lipids, the optical properties of cyanine dyes like IcG can also be modified through J-aggregation.^62^ J-aggregation is a simple process where molecules at high concentration undergo stable “head-to-tail” stacking leading to the formation of large supramolecular structures. Notably for IcG, this process results in a nearly ~100nm redshift in the peak absorption, an increase in absorption magnitude, and an increase in photostability relative to monomeric IcG.^60^,^63^-^69^ J-aggregates exhibit enhanced optical properties at unique wavelengths, are responsive to environmental changes, and can be tailored for specific theranostic purposes.^68^,^70^,^71^ For example, it has been shown that highly soluble DCP-Cy J-aggregates could be encapsulated in liposomes enabling efficient fluorescence and photoacoustic imaging.^72^ Recently it has also been demonstrated that enzymatic collapse of J-aggregate containing liposomes in the treated tumor microenvironment restores fluorescence activity creating a self-reporting dye.^73^ With peak absorption at roughly ~890nm, IcG J-aggregates are a highly absorbent and biodegradable particle at a wavelength typically dominated by non-degradable metallic substances like gold. Recently, our group demonstrated that IcG J-aggregates could be encapsulated within liposomes using a facile and reproducible method^60^, however the use of these liposomal IcG J-aggregates (LJA) as an effective absorber for both PAI and PTT has not been extensively explored.

Unlike IcG in its soluble form, IcG J-aggregates respond well to sustained or repeated illumination for heat generation.^74^,^75^ This improved photostability is a major benefit of J-aggregates as lengthy and repeated heating are often necessary to achieve therapeutic effects. Beyond improved photostability, the redshift in absorbance following J-aggregation of IcG offers a unique opportunity for further investigation for PTT. As mentioned previously, most studies involving the use of IcG for PTT utilize laser diodes with wavelengths of ~808nm both due to the peak absorbance of IcG in blood and the widespread availability of these lasers.^18^,^52^-^54^,^74^-^76^ While 808nm lasers are still effective for PTT with IcG J-aggregates^65^,^77^, longer wavelength lasers closer to its peak absorption of 890nm would improve heat generation and penetration depth due to lower attenuation and scattering from endogenous agents such as blood and water.^73^,^78^-^81^ Crucially, the use of longer NIR-I wavelengths has not been extensively explored for PTT with J-aggregates despite the apparent advantage over 808nm diodes. As noted previously, a wide variety of agents have been explored for PAI and PTT; however, compared to other leading candidates such as gold nanoparticles, LJA offer several key advantages. These include well-characterized clearance and biodegradation pathways for all major components, reliance on FDA-approved materials that are already in widespread clinical use, and a facile, low-cost, and reproducible synthesis that can be readily modified for specific applications such as active targeting or drug delivery. Therefore, it is the goal of this study to not only demonstrate the superior performance of LJA over monomeric IcG for PAI and PTT in a cancer model, but also to show the utility of slightly longer wavelength lasers in PTT studies involving LJA.

## 2. Results and Discussion

**Figure 1:**
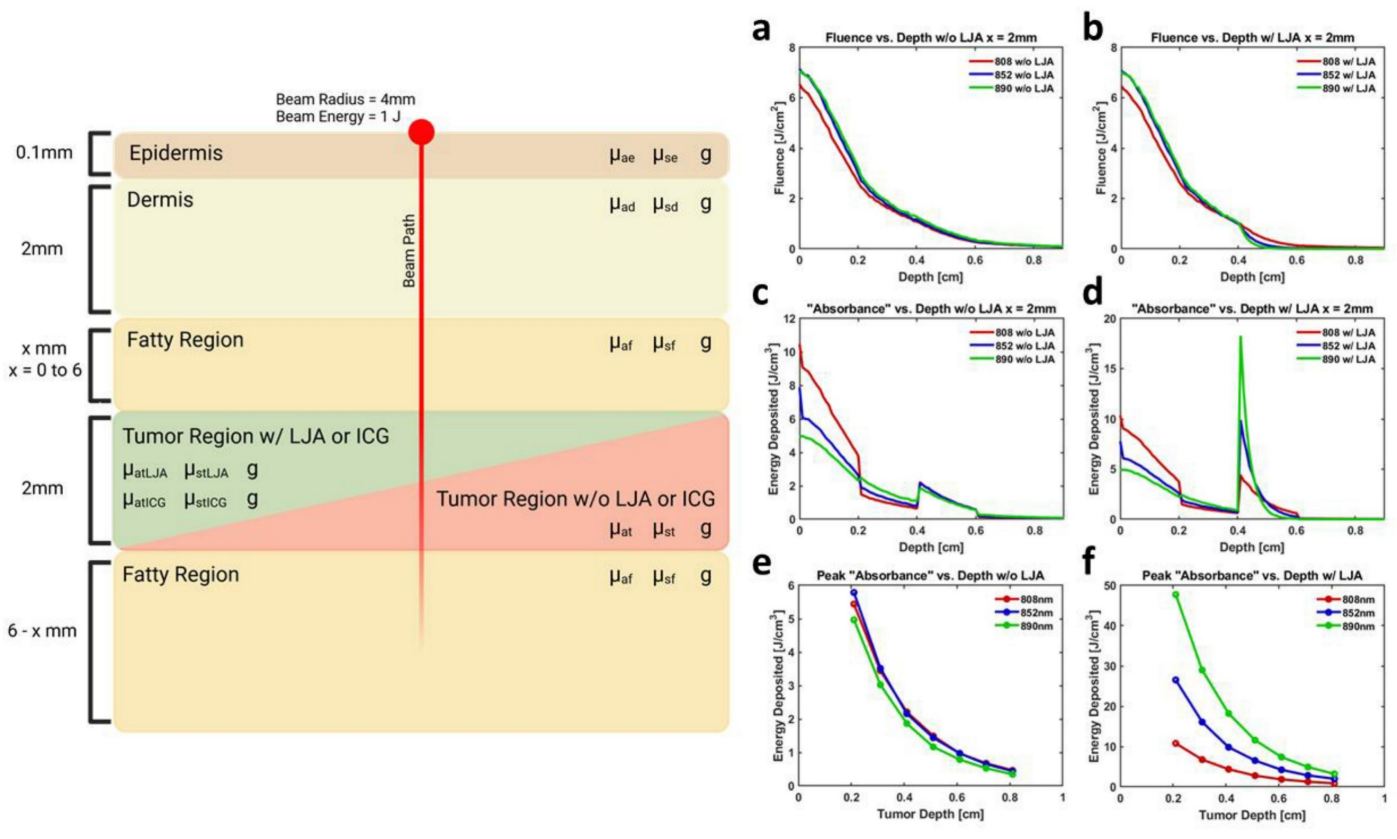
**Monte Carlo Simulation of Light Incident on Multilayered Tissue.** Schematic depicts representative geometry of the multilayered system. Tissue is modeled with layers representing the epidermis, dermis, fat, and tumor with or without contrast agents. Each layer has unique values relevant to the optical path: *μ*_a_, optical attenuation, *μ*_s_, optical scattering, and *g*, anisotropy factor. Values obtained from literature 78,79 (Figure S2). Dimensions are as indicated, with the tumor region being placed at variable depths below a fat layer of length *x*. (a) Light fluence vs depth of system containing tumor under 2mm of fat without LJA and (b) with LJA. (c) Absorption vs depth of system containing tumor under 2mm of fat without LJA and (d) with LJA. (e) Peak absorbance at tumor interfaces of varying depths in tissue without LJA and (f) with LJA.

**Figure 2:**
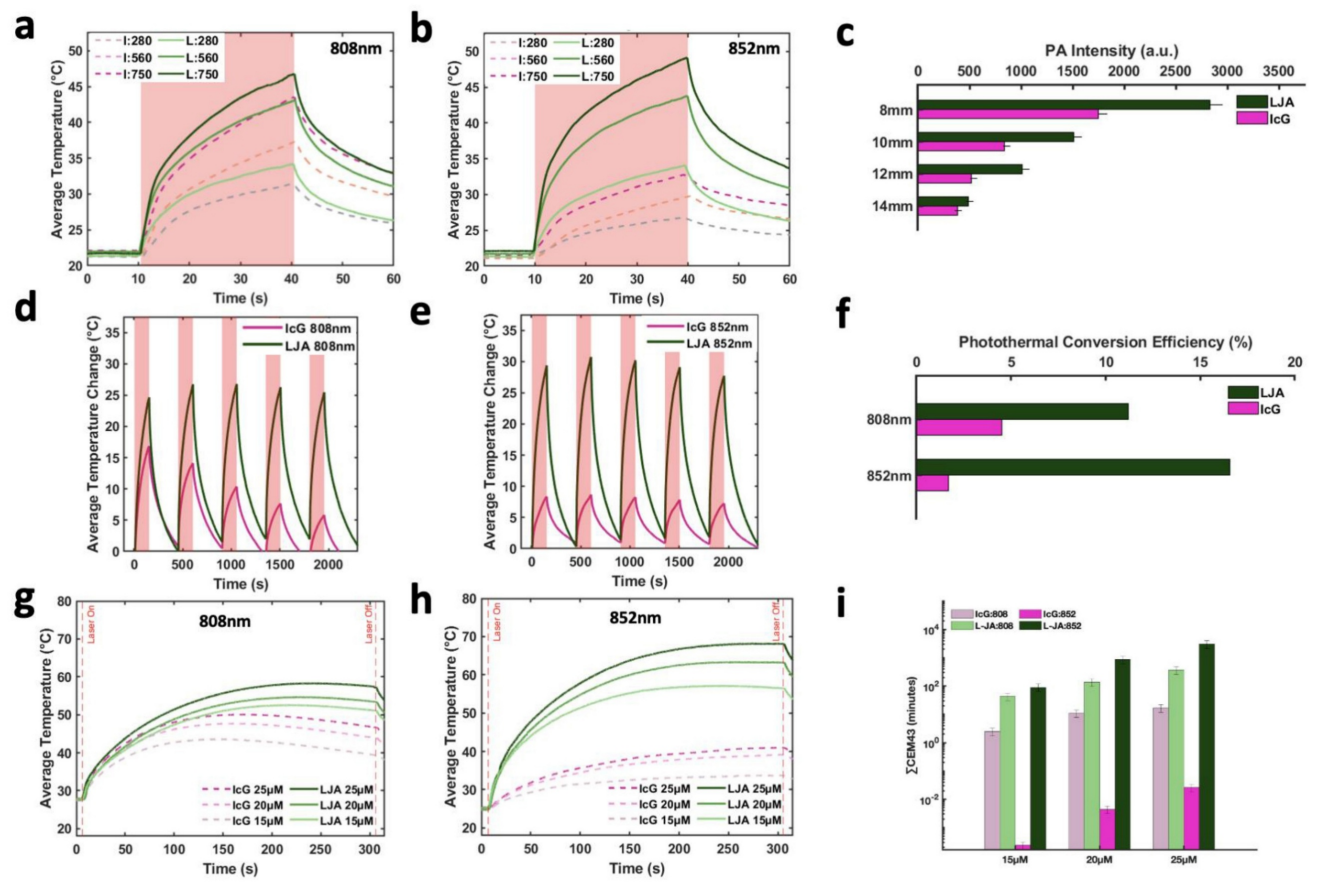
**Characterization of LJA and IcG for PTT.** (a,b) Average temperature of solution while undergoing 30s of laser irradiation at (a) 808nm and (b) 852nm at three different power levels (280, 560, and 750 mW/cm^2^). (c) Photoacoustic signal generation at depth in tissue mimicking phantom for 780nm (IcG) and 890nm (LJA) illumination. (d,e) Average change in temperature of solution under repeated cycles of heating at (d) 808nm and (e) 852nm. Irradiation for 150s followed by cooling for 300s. Studies conducted at 20μM. (f) Photothermal conversion efficiency (PCE) of LJA and IcG at 808nm and 852nm. (g,h) Average temperature of solution during 300 seconds of laser irradiation for (g) 808nm and (h) 852nm. Values obtained for three different solution concentrations (15μM, 20μM, and 25μM). Studies conducted at 750 mW/cm^2^. (i) Thermal dose calculated as the cumulative equivalent minutes at 43°C (CEM43). Increasing concentration equates to a higher thermal dose in phantoms.

LJA were synthesized as previously reported.^60^ Liposomes synthesized for this study were found to have an average diameter of ~130nm, average zeta potential of -27.2mV, and peak optical absorption and photoacoustic signal generation near 890nm (Figure S1). As mentioned previously, 808nm laser diodes are typically employed for PTT of IcG; however, the redshift in J-aggregate absorption suggests longer wavelengths may be more effective. To begin the investigation of that claim and to approximate how these particles would respond *in vivo,* a Monte Carlo simulation was conducted to model light penetration and absorption of 808nm, 852nm, and 890nm wavelengths through multi-layered biological tissue containing a tumor and LJA. The well-documented MCML and CONV programs made public by Lihong Wang and Steven Jacques were used for this study.^82^,^83^ A simplified tissue model was designed to include homogenous layers of skin, fat, and tumor, each with unique optical properties including optical attenuation factor, *μ*_a_, optical scattering factor, *μ*_s_, and tissue anisotropy factor, *g* (Figure 1a). The MCML program takes these parameters and simulates a fluence distribution in terms of depth and radial distance from the photon entry point using a weighted “random walk” calculation. The CONV program takes the fluence profile generated by MCML and convolves it with a gaussian finite beam profile to generate a map more representative of a realistic beam shape. To investigate how properties changed with depth, the simulation was performed multiple times with the tumor region under a layer of fat with thickness *x* varying from 0mm to 6mm. Values for optical properties were obtained from published literature (Figure S2).^78^,^79^,^84^,^85^ Estimates for *μ*_a_ and *μ*_s_ in tumors containing LJA were made assuming <~1% of an injected dose of 5mg/kg was uniformly distributed through the region. With this assumption, estimates were calculated via linear combination of known values for tissue and particles.^86^,^87^ The resulting values for fluence and absorbance at depth serve as a good first approximation for how these wavelengths of light would interact with tumor bearing tissue. From Figure 2a, c, and e, in the absence of LJA, fluence and absorbance for each wavelength are nearly indistinguishable at most depths. However, the introduction of LJA in Figure 1b, d, and f, leads to significant heating at deeper depths for 852nm and 890nm light compared to 808nm light. While fluence changes are minimal, the increased absorbance of LJA at 852nm and 890nm leads to more than 3 times the energy deposition than at 808nm. As both photoacoustic signal generation and temperature changes can be directly related to light absorption in tissue, these results suggest that wavelengths closer to 890nm would be most effective for PAI and PTT involving LJA.

To provide quantitative evidence to support estimates from the Monte Carlo model, *in vitro* experiments were conducted to compare IcG- and LJA-mediated imaging and heating. Initial work was conducted to compare photoacoustic and photothermal properties of LJA and IcG as well as how they respond when obstructed by tissue mimicking material. Using tissue mimicking polyacrylamide phantoms loaded with LJA or IcG, the photoacoustic signals generated by the two agents were investigated at different depths. Concentrations were matched between samples to enable more accurate comparison. Signal diminishes with depth as both optical and acoustic signal is attenuated by phantom material, but signal intensity of LJA with 890nm illumination (Figure 2c) is significantly higher than intensity of IcG at 710nm (Figure S3) and 780nm (Figure 2c) at all depths investigated. Ideally, heating investigations using a corresponding 890nm laser diode for PTT with LJA would be conducted; however, available diodes at this wavelength do not provide sufficient power for PTT and larger laser systems are not favorable due to prohibitive cost and larger footprint. As such, further investigation is conducted with more readily available 808nm and 852nm laser diodes. At 808nm, equal concentrations and laser powers of LJA and IcG show similar levels of heating (Figure 2a and 2g) while at 852nm, solutions containing LJA reach much higher average temperatures (Figure 2b and 2h). Under repeated illumination cycles at 808 nm, LJA show higher levels of photostability while IcG degrades and loses its ability to generate heat (Figure 2d). This trend continues at 852nm as LJA maintain their ability to generate heat under repeated cycles of illumination (Figure 2e). Interestingly, IcG does not appear to degrade upon repeated heating at 852nm, but overall heat generation is minimal.

Photothermal conversion efficiency (PCE) for LJA and IcG were also calculated using the equation,



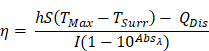



as described by similar reports^88^,^89^. Briefly, PCE, or *η*, is calculated as a ratio of temperature change in the solution relative to the laser power and magnitude of absorbance at the wavelength of interest. In this equation, *h* is the heat transfer coefficient, *S* is the surface area of the container, *T_Max_* is the maximum temperature reached, *T_Surr_* is the surrounding temperature, *Q_Dis_* is heat dissipated from a cell containing only the background solution, *I* is laser power incident on the sample, and *Abs_λ_*, is absorbance at wavelength *λ*. The value of the product *hS* is calculated from



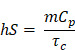



where *m* is mass of the entire phantom material, *C_p_* is the heat capacity of water, and *τ_c_* is the heat transfer time constant for the system. *τ_c_* is obtained from the linear curve of cooling time versus ln(*θ*) (Figure S4 and S5) where



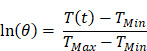



At 808nm, the PCE for IcG is 4.6% while for LJA it is 11.8%. At 852nm PCE for IcG drops to just 1.8% while for LJA it climbs to 17.1% (Figure 2f). Higher PCE at a given wavelength indicates that at equal concentrations, LJA would more efficiently transfer light energy to heat. Finally, to quantify thermal dose *in vitro,* cumulative equivalent minutes at 43°C (CEM43) was calculated as reported elsewhere using the equation



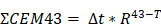



where *∆t* is the time interval, *T* is the temperature, and *R* is a constant; 0.25 if T < 43°C and 0.5 if T > 43°C.^90^,^91^ PTT with LJA provides higher thermal dose than IcG at both 808nm and 852nm (Figure 2i). In comparison to IcG at 780nm, the proportionally larger PAI signal generated by LJA at 890nm (Figure 2c) can be attributed to a combination of increased absorbance of LJA at this wavelength and a higher proportion of energy transferred to heat in J-aggregate forms.

**Figure 3:**
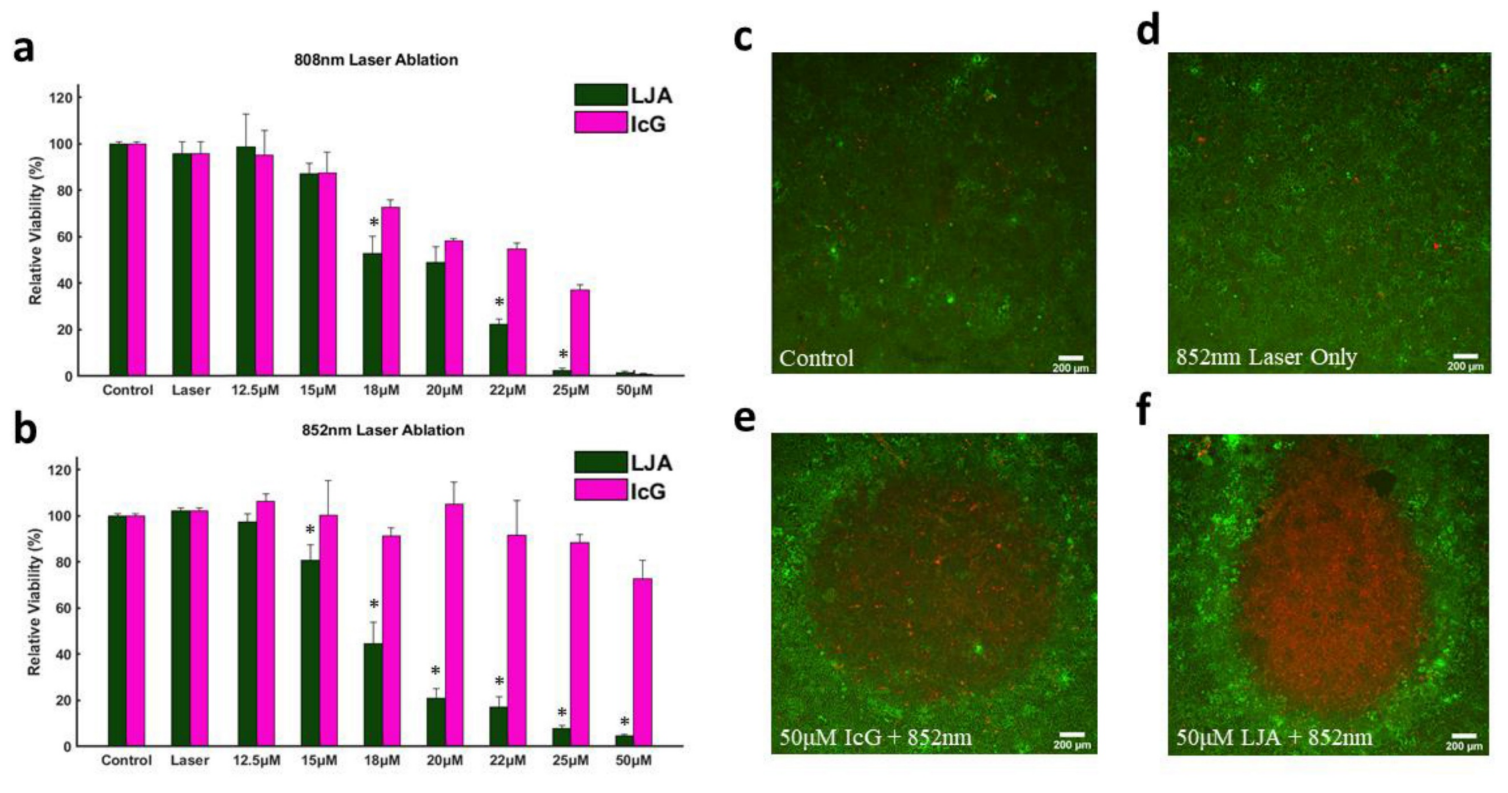
***In Vitro* Photothermal Toxicity at 808nm and 852nm.** (a,b) Relative viability of KPC cells as determined by CCK-8 assay following 300s of PTT at 750mW/cm^2^ with varying concentrations of LJA and IcG for (a) 808nm and (b) 852nm (p<.05). All LJA and IcG samples above 18μM are significantly different than Control and Laser only groups for 808nm. All LJA samples above 15μM are significantly different than Control and Laser Only groups for 852nm treatment. (c-f) Calcein AM/EthD-III staining of cells immediately after 60s of 750mW/cm^2^ treatment with 852nm laser. Green represents living, red represents dead. (c) Control (no treatment) (d) Laser Only (0μM of LJA or IcG), (e) IcG (50μM) and (f) LJA (50μM).

**Figure 4:**
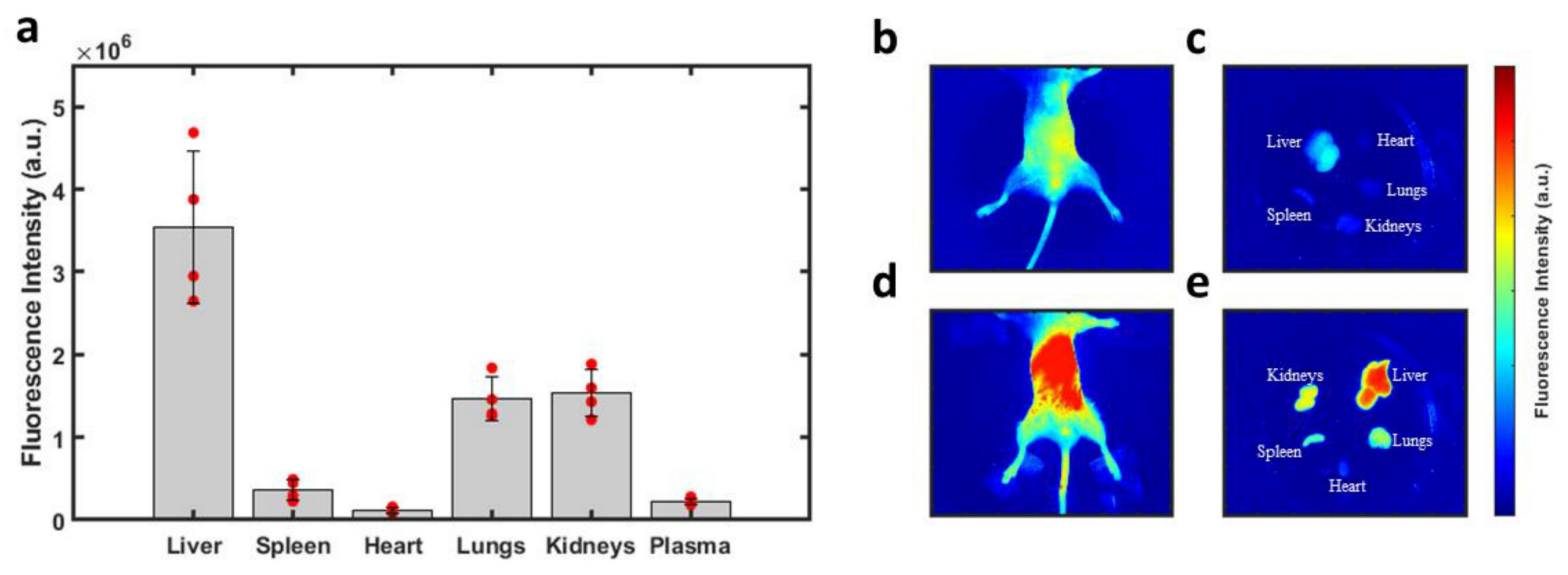
** Biodistribution of Injected Nanoparticles.** (a) Fluorescence intensity observed in perfused organs 24 hours after injection of liposomal IcG contrast agent. Intensity is normalized by tissue weight and accounts for background fluorescence from control tissue. No other organs exhibited significant uptake of the nanoparticles. Major uptake/clearance occurs in the tissue of the reticuloendothelial system (RES). (b) Whole body fluorescence of a control mouse and (c) its perfused organs. (d) Whole body fluorescence of a mouse which received liposomal injection and (d) its perfused organs 24 hours after injection. (n=4) All error bars depict S.D.

Next, the capacity to photothermally induce cell death with IcG and LJA was investigated in the mT4-2D strain of KPC cells. KPC cells were seeded in 96-well plates and allowed to adhere for 24 hours before cell culture media was replaced with media containing different concentrations of LJA and IcG (0μM, 12.5μM, 15μM, 18μM, 20μM, 22μM, 25μM, and 50μM). Immediately after replacing the media, wells were exposed to 300s of 750 mW/cm^2^ laser irradiation at 808nm or 852nm. After heating, media was replaced with fresh culture media and cells were again allowed to incubate for 24 hours before viability was assessed via CCK-8 assay. As shown in Figure 4a and 4b, LJA is significantly more effective as a PTT contrast agent than IcG at several concentrations under 808nm irradiation and every concentration under 852nm irradiation. To better visualize this effect, in a similar study KPC cells were seeded in a 24-well plate and allowed to grow to ~90% confluency. Media was replaced with 50μM of LJA or IcG before 1 minute of 750mW/cm^2^ irradiation at 852nm. To visually depict the area of immediate effect, the media was immediately replaced, and cells were stained with Calcein AM/EthD-III to show living (green) and dead (red) cells (Figure 3c- f).

With confirmation that PTT with LJA is superior to IcG at 808nm and 852nm *in vitro*, the focus then shifted to testing the performance of LJA as a cancer theranostic agent *in vivo*. It is well-known that IcG clears rapidly from blood circulation over the course of several minutes. From blood it collects in the liver before rapid clearance into bile. IcG is virtually non-existent in the body after 24 hours, with full clearance likely occurring within hours (Figure S6).^92^-^94^ As liposomes prolong circulation, it was hypothesized that LJA would last significantly longer than monomeric IcG and would undergo different clearance pathways. To test this, a study to characterize the uptake and biodistribution of the injected nanoparticles was performed. While inside a whole-body fluorescence imaging system, albino C57BL/6 mice (n=4) received a bolus 5mg/kg dose of contrast agent via the tail vein before sacrifice and tissue harvest 24 hours later (Figure 4). As J-aggregation significantly reduces the fluorescence of IcG, liposomes containing IcG that had not fully aggregated were used for fluorescence imaging. Major uptake/clearance occurs in tissues of the reticuloendothelial system (RES), including the liver, spleen, and kidney (Figure 4e). Control mice (Figure 4b and 4c) show minimal fluorescence in all tissues. Notably, after 24 hours, while most of the contrast agent has been cleared from circulation, there is still substantially more fluorescence signal in the plasma of mice that received liposomal formulations than those that received monomeric IcG (Figure S6).

**Figure 5:**
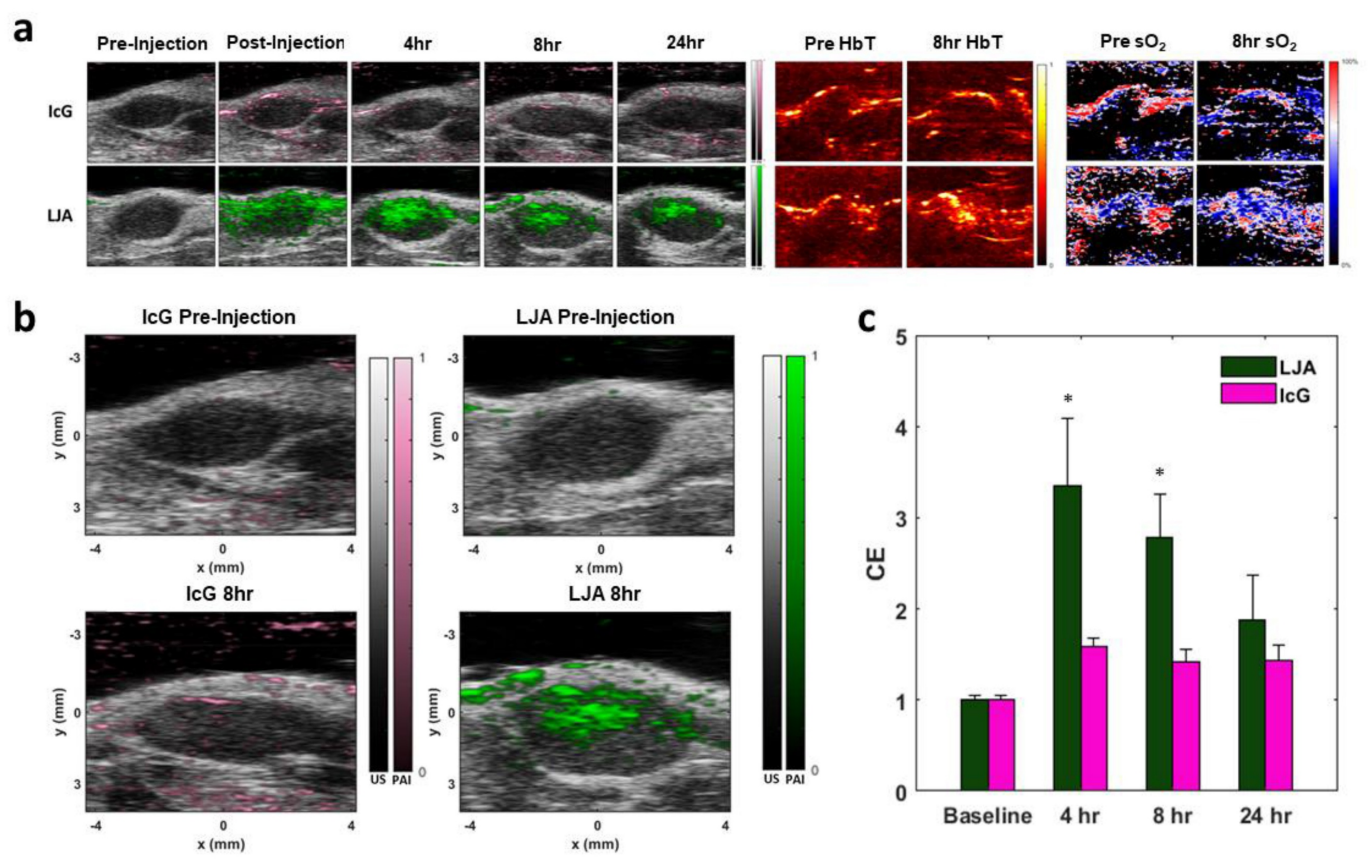
***In Vivo* KPC Tumor Imaging.** (a) Sample photoacoustic images showing contrast agent signal, total hemoglobin (HbT), and estimates of oxygenation (sO_2_). Top row shows PA intensity overlaid on B-Mode images of tumors before and after IcG injection, as well as HbT and sO_2_ before and 8 hours after injection. Bottom row shows PA intensity overlaid on B-Mode images of tumors before and after LJA injection, as well as HbT and sO_2_ before and 8 hours after injection. (b) Higher magnification images of PA signal before injection and 8 hours after injection for IcG (left) and LJA (right). (c) Contrast enhancement (CE) provided by LJA and IcG at 4 hours, 8 hours, and 24 hours after injection relative to baseline. CE calculated as the ratio of unmixed contrast agent signal at time point to the unmixed contrast agent signal prior to injection. (n=4) (p<.05).

Next, the extravasation and accumulation of IcG and LJA in tumors was investigated *in vivo*. The same strain of KPC cells used for *in vitro* investigation were injected subcutaneously in the hind flanks of albino C57BL/6 mice. Tumors grew over the course of two weeks before reaching an optimal size for imaging and treatment. To monitor tumor uptake of both LJA and IcG and to determine an optimal time for PTT, each mouse received a 5mg/kg intravenous dose of either LJA, IcG, or saline control. The entire tumor region was imaged using a 3D motor prior to injection (baseline) and at *t* = 0hrs, 4hrs, 8hrs, and 24hrs after injection. Tumors were illuminated at multiple wavelengths within the spectra of hemoglobin, IcG, and LJA and acoustic emissions were received by the ultrasound imaging probe. Data was spectrally unmixed using Visualsonics software and images of IcG or LJA distribution, total hemoglobin, and estimated blood oxygen levels were constructed (Figure 5a). Contrast enhancement (CE) was calculated as a ratio of signal before injection to signal at the time point of interest, and it demonstrates that LJA provide significantly higher signal than IcG following injection (Figure 5b,c). Signal in the tumor region more than doubles for LJA at all time points investigated. OxyHemo imaging, which provided values for total hemoglobin (HbT) and an estimate for blood oxygen saturation (sO_2_) do not indicate a shift in measured oxygen content following injection (Figure 5a, S6, and S7).

**Figure 6:**
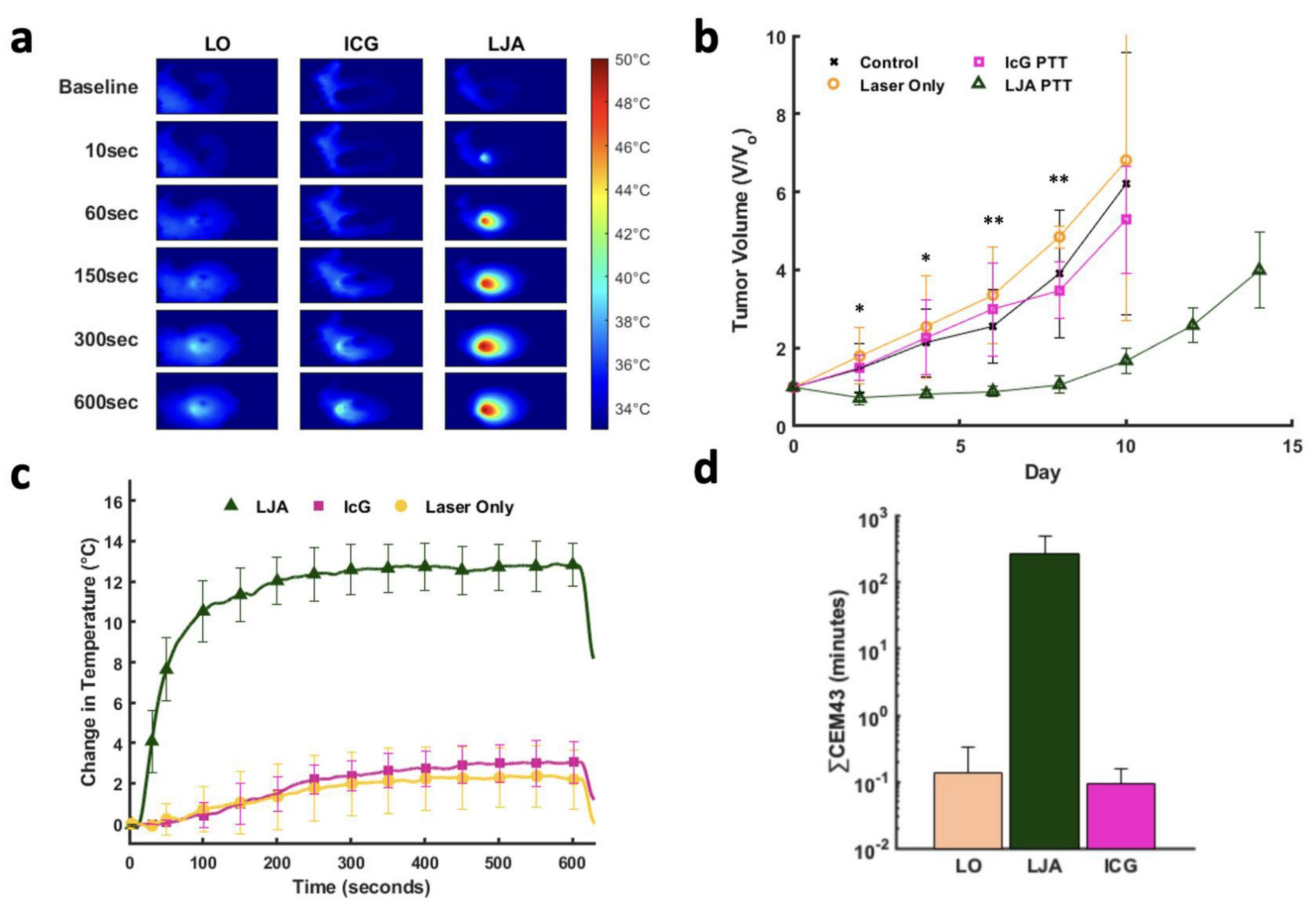
** PTT of Subcutaneous KPC Tumors at 852nm.** (a) Heat maps of mouse tumors for Laser Only (LO), IcG, and LJA treated groups prior to laser exposure (baseline) and after 10, 60, 150, 300, 600 seconds of 852nm laser irradiation. (750mW/cm^2^). (b) Relative tumor volume for control (black), LO (orange), IcG (pink), and LJA (green) groups. LJA treatment results in arrested growth for several days, while the other groups show no change in tumor growth. (c) Average change in temperature of the tumor region during treatment for LO, ICG, and LJA groups. (d) Thermal dose for each treatment group measured as CEM43. (n=5) (*=p<.05, **=p<.01) All error bars depict S.D.

Next, PTT was conducted in a separate cohort of tumor bearing mice 8 hours following injection of contrast agent. Mice were separated into four groups, LJA, IcG, Laser Only (LO), and control. Eight hours was selected for PTT as this timepoint showed appreciable accumulation of both LJA and IcG and is long enough after injection to ensure most of the injected contrast agents had left circulation either by clearance through the RES or accumulation in tumor tissue. *In vivo* PTT was performed at 750mW/cm^2^ for 10 minutes to mirror *in vitro* studies. Therapy was conducted once tumors reached roughly 50mm^3^ in volume and mice were monitored every other day. Per IACUC instructions, mice were euthanized if tumors exceeded 500mm^3^ or if tumor growth led to ulcerations on the mouse skin. Relative to IcG, the higher absorbance and tumor accumulation of LJA makes them more effective for PTT at 8 hours post injection. Mice receiving LJA showed significantly higher temperature changes in the tumor region than both IcG and LO mice (Figure 6a,c). Following treatment, mice were monitored for two weeks. Body weight and tumor size measurements were performed at specific time points every other day (Figure S9). By day 10, less than 2 mice survived in the control, LO, and ICG+PTT groups while all LJA+PTT mice reached day 14. Mice in the LJA+PTT group show a significant drop off in tumor growth following treatment when compared to IcG+PTT, LO, and control groups (Figure 6b). In fact, PTT with LJA appears to arrest tumor growth temporarily. Under this treatment regime, remission is temporary as indicated by the resurgence in tumor growth after day 8. Nevertheless, the use of higher laser powers and LJA concentrations or conducting PTT sooner after injection could help achieve complete ablation as shown in other reports.^64^,^65^ However, the use of higher temperatures does increase the risk of more serious off-target burns and potential ulceration, so it is necessary to balance therapeutic effect and adverse effects. To further quantify the therapeutic effect and thermal dose of PTT, CEM43 was calculated for LJA, IcG, and LO groups. CEM43 was calculated as reported previously. It should be noted that while the treated tumors themselves are 3-dimensional, thermal measurements are only collected via camera at the surface of the tumor. As such, the true thermal dose experienced across the tumor is likely different as certain regions with higher or lower contrast agent concentration would experience localized temperature changes. Nevertheless, it is clear that LJA-treated mice receive a significantly higher thermal dose than mice in LO and IcG-treated groups. (Figure 6d). Optimization of thermal dose through adjustments in timing, concentration, and laser power offers the versatility to target hyperthermic or ablative temperature levels as desired.

## 3. Conclusion

Liposomal J-aggregates of Indocyanine Green (LJA) offer a unique contrast agent that is biodegradable, utilizes FDA-approved components, and provides significant advantages over conventional IcG in terms of circulation time, photostability, tumor uptake, and active NIR wavelengths. With liposomal encapsulation extending circulation time, and the importance of the redshifted peak absorbance demonstrated with 890nm PAI and 852nm PTT, LJA are more versatile than other conventional optoacoustic contrast agents. Continued investigation of their potential at longer wavelengths and greater depths is necessary to further demonstrate their advantages and clinical potential. Currently, optical approaches are heavily limited by tissue attenuation, meaning they lose their efficacy in deeper tissues. The benefits offered by LJA, specifically an elevated response at longer wavelengths, begin to overcome this barrier and makes them a more attractive option as a potential theragnostic agent, both on their own and in combination with other techniques. Given that LJA absorbance peaks at 890 nm, there is potential to continue lowering laser power levels for safer exposures to intervening tissues during PTT. Future work that demonstrates the benefit of PTT with LJA as a co-therapy in more clinically relevant cases may be an attractive avenue for continued development of PTT as a promising technique.

## 4. Materials and Methods

### 4.1. Materials

Indocyanine Green (IcG) was purchased from Chem-Impex (Wood Dale, IL, USA). 1,2-diarachidoyl-sn-glycero-3-phosphocholine (20:0 PC), 1,2-distearoyl-sn-glycero-3-phosphoethanolamine-N-[methoxy(polyethylene glycol)-2000] (DSPE-PEG2000), and cholesterol were purchased from Avanti Polar Lipids (Birmingham, AL, USA). Chloroform, ethanol, ammonium persulfate (APS), N,N,N',N'-Tetramethylethylenediamine (TEMED), and titanium oxide (TiO_2_), were purchased from Sigma-Aldrich (St. Louis, MO, USA). 40% 19:1 acrylamide/bisacrylamide was obtained from Research Products International (Mt. Prospect, IL, USA). Black India ink was purchased from Chartpak (Leeds, MA, USA). CCK-8 Assay was purchased from APExBIO (Houston, TX, USA). Live/Dead Assay containing Calcein AM/EthD-III purchased from Biotium (Fremont, CA, USA).

### 4.2. IcG Calibration Curve

Indocyanine Green was dissolved in a 1:1 (v/v) solution of ethanol and ultra-pure deionized water to achieve an initial concentration of 10mM. To ensure proper dissolution, IcG was vortexed and sonicated in intervals for at least 1 hour and allowed to rest 30 minutes prior to continued work. The IcG/Water/Ethanol solution was serially diluted 9 times to a concentration of 0.02mM. Using a NanoDrop Spectrophotometer (Thermo Scientific, Waltham, MA, USA), UV-VIS absorbance spectra for each dilution were collected. The entire process, starting with a fresh 10mM IcG solution was repeated four times. Raw values for the 780nm absorbance peak, specific to monomeric IcG, were averaged and used to generate plots of absorbance intensity versus concentration. A linear relationship was observed up to a concentration of 2mM. From this linear relationship, a curve was generated to enable comparing solutions within and across studies. To measure IcG content, all particle solutions were combined in a 1:1 ratio with ethanol and compared to this curve.

### 4.3. J-Aggregate Liposome Synthesis

20:0 PC, cholesterol, and DSPE-PEG2000 dissolved in chloroform were combined in a 9/4/1 molar ratio in a 4mL glass vial. Chloroform was slowly evaporated over the course of 5 to 10 minutes under a steady stream of argon gas until a thin film developed at the bottom of the vial. The vial was then placed inside a larger glass vessel connected to a Rotavapor R-300 rotary evaporator (Buchi, Berlin, Germany). The rotary evaporator was then slowly brought down to a pressure setting of 80mBar before allowed to sit undisturbed for at least 2 hours to ensure all solvent was removed. 2ml of a 2.5mM IcG solution in ultra-pure deionized water is added to the vial to rehydrate the lipid film. The solution was briefly heated to 80°C and cyclically vortexed to allow for complete hydration of the lipid film. The hydrated glass vials were then sonicated in a bath sonicator (Fischer Scientific, Hampton, NH, USA) for ~20 minutes to complete the formation of liposomal vesicles.

Next, this solution was passed through 100nm pore-size polycarbonate membranes (Avanti Polar Lipids) 11 times using an extruder (Avanti Polar Lipids) and two glass syringes (Hamilton, Reno, NV, USA). To remove excess IcG which was not encapsulated in the liposomes, the solution was then dialyzed using 300kDa dialysis tubing for 24 hours (Fischer Scientific). DI water was replaced every 8 to 12 hours. J-Aggregation of encapsulated IcG was completed by heating the liposomal solution in a 60°C heating block (Fischer Scientific) for 24 hours. To ensure aggregation had been completed, the UV-VIS spectra of the solution were obtained using a UV-VIS-NIR Spectrophotometer (Shimadzu, Kyoto, Japan). Final particles were concentrated via centrifugation with 100kDA filter units (Sigma-Aldrich) and resuspended in 1X phosphate-buffered saline for storage at 4°C. Particles were used within 2 weeks of synthesis. Size and zeta potential were measured using a Zetasizer (Malvern Panalytical, Malvern, UK). Particles were diluted by 1000x in 1X PBS for measurement. Low volume polystyrene cuvettes were used for size measurements whereas standard zeta cells were used for zeta potential measurements.

### 4.4 Monte Carlo Simulation

The MCML and CONV models made public by Lihong Wang and Steven Jacques were used for this study. Full documentation and recommendations are available here https://omlc.org/software/mc/. Briefly, the models allow for the creation of one-dimensional layers of tissue, each with their own optical properties. The major tissue parameters include optical attenuation, optical scattering, and tissue anisotropy. Additional input parameters include number of photons, grid resolution, and grid size. With the suggested geometry and optical parameters, the MCML model simulates the path of an infinitesimally small beam of photon packets as they are either absorbed or scattered along their path through the pre-defined tissue layers. Photon movement is simulated as a “random walk” where a random distance between interactions is calculated using an exponential distribution defined by the attenuation and scattering properties of the material. Potential outcomes of each interaction are either photon absorption or photon scattering in a new direction informed by the tissue anisotropy factor. In essence, the higher the attenuation or scattering coefficients are, the more likely an event will occur. To reduce overall computation intensity, the MCML model relies on a simple weighting system where each photon packet can be used to simulate many different potential outcomes. Starting with a weight of 1.0, after each event a fraction of the photon's weight - corresponding to the absorption coefficient of that layer - is deposited. The path of the photon will continue to be simulated until the weight decreases below a termination threshold, all photons are absorbed, or all photons exit the medium.

The CONV model takes the output of the MCML simulation and convolves it with a defined beam profile to produce a more realistic multi-dimensional result. Moving radially out from the point source used for the MCML model, the CONV model can generate more realistic beam profiles and estimations for photon fluence and absorption through a two-dimensional space. To model the impact of laser wavelength on PTT efficacy, a tissue construct defined by 0.1mm epidermal, 2.0mm dermal, 6.0mm fatty, and 2.0mm tumor regions was input into the MCML model. 107 photons were simulated with depth step sizes of 10 microns and radial step sizes of 25 microns. Photon path results were convolved with a 1 Joule, 4mm radius gaussian beam. To model how the depth of the tumor region impacted fluence and heat generation, the geometry was altered between runs such that the tumor was present at different depths. Simulations were run using values consistent with 808nm, 852nm, or 890nm light. Comparisons were made between untreated tumors, IcG-treated tumors, and LJA-treated tumors by assuming an equal concentration of each agent was uniformly spread across the tumor region. In this work parameters for optical attenuation, scattering, and anisotropy were obtained from literature reports. For tissue sections containing contrast agent, the values for attenuation and scattering were calculated as a linear combination.

### 4.5. *In Vitro* Photoacoustic Imaging

*In vitro* photoacoustic tests were performed using clear polyethylene tubing (Roboz Surgical Instruments, Gaithersburg, MD, USA) embedded within tissue-mimicking polyacrylamide phantoms. Phantoms were created by following the approach of Hariri et. al.^95^ Phantom precursor consists of a 12% w/v acrylamide solution containing APS (0.08% w/v), black ink (0.004% v/v) and TiO_2_ (0.08% w/v). Precursor was degassed under vacuum with frequent argon purges for 1 hour. To initiate polymerization, TEMED was added at 0.2% v/v. Following addition of TEMED, the rapidly polymerizing solution was poured in molds where they could fully solidify and cool over the course of an hour.

All photoacoustic imaging was performed using the VEVO LAZR-X and VEVO 2100 systems (FUJIFILM VisualSonics, Toronto, Canada). Calibrations were performed using provided calibration equipment at the beginning of each day. For depth dependent imaging, tubes embedded within the phantoms were filled with LJA or IcG. The transducer was coupled to the phantom using clear ultrasound gel to allow imaging to occur. PA spectra were gathered to determine the wavelength of maximum signal generation, 780nm for IcG, and 890nm for LJA. Images were acquired at each of these wavelengths allowing for signal intensity to be captured from each tube.

### 4.6. Photothermal Heating

All laser components were acquired from ThorLabs (Thorlabs Inc., Newton, NJ, USA) Both an 808nm and an 852nm laser diode, were mounted, aligned, and focused in the range of an IR camera. (Teledyne FLIR, Wilsonville, OR, USA). Optical setups were identical other than the addition of an optical isolator (ThorLabs) as recommended by the manufacturer to limit damage to the diode. Laser power was calibrated to 750 mW/cm^2^ for both lasers and across all experiments. All experiments were conducted in 24- or 96-well plates or in a polystyrene cuvette filled with corresponding contrast agent solutions placed at the focus of the laser and IR camera.

As discussed previously, photothermal conversion efficiency (PCE) for LJA and IcG were also calculated using the equation,



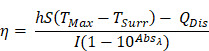



as described by similar reports.^88^,^89^ h is the heat transfer coefficient, S is the surface area of the container, T_Max_ is the maximum temperature reached, T_Surr_ is the surrounding temperature, Q_Dis_ is heat dissipated from a cell containing only the background solution, I is laser power incident on the sample, and Abs_λ_, is absorbance at wavelength *λ*. The value of hS is calculated from



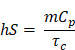



where m is mass, C_p_ is the heat capacity of water, and τ_c_ is the heat transfer time constant for the system. τ_c_ is obtained from the linear curve of cooling time versus ln(*θ*) (sup) where



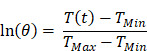



### 4.7. *In Vitro* Photothermal Therapy

All *in vitro* PTT experiments were conducted using the mT4-2D variant of KPC cells. These cells were cultured in high glucose Dulbecco's modified Eagle medium (DMEM) supplemented with 10% (v/v) fetal bovine serum and 1% penicillin-streptomycin. Cells were incubated at 37 °C with 5% CO_2_ in a humidified atmosphere. To quantify the PTT effect, KPCs were seeded at the density of 20,000 cells per well in 96 well plates. After 24 hours, the culture media was replaced with pre-warmed fresh media containing L-JA or IcG at the desired test concentration. Cells incubated without particles were used as a control group. Plates were then immediately brought into the laser path and exposed to 750 mW/cm^2^ of either 808nm or 852nm light for 5 minutes. Following treatment, media was again replaced, and cells were allowed to rest for 24 hours. Finally, the CCK-8 assay was performed per manufacturer instructions. Briefly, 100uL of fresh media and 10uL of assay solution were added to each well and incubated for 4 hours. Plates were then gently agitated to remove bubbles and uniformly disperse the supernatant before the absorbance signal at 490nm was measured using a BioTek plate reader.

Qualitative images of laser treated cells were obtained using cells seeded in 24-well plates. KPCs were seeded under the same parameters and allowed to grow to near full confluence. Media was replaced with LJA or IcG containing media and wells were immediately irradiated under 852nm light at 750 mW/cm^2^ for 2 minutes. Media was immediately replaced, and cells were stained with Calcein AM/EthD-III to show living (green) and dead (red) cells. Images captured using a Leica Fluorescent Microscope (Nussloch, Germany).

### 4.8. Subcutaneous Tumor Implantation

All *in vivo* experiments were conducted using protocols approved by the Institutional Animal Care and Use Committee at the University of Texas at Austin. All experiments were performed on albino 6-9 weeks-old mice from Jackson Laboratory (Bar Harbor, ME, USA). Anesthesia was induced using 5% isoflurane in an induction chamber. Once unconscious, anesthesia was maintained with 2-2.5% isoflurane. The right hind flank of the mouse was shaved and cleaned to obtain a clear injection site for the cells. 3.5*10^5^ KPCs suspended in PBS were injected subcutaneously in each mouse before allowing the mice to return consciousness. Mice were monitored frequently over the course of two weeks to allow the tumors to stabilize and grow to an optimum size (roughly 50mm^3^) prior to imaging or treatment.

### 4.9. *In Vivo* Imaging and Tumor Uptake

Tumor-bearing mice were anesthetized and shaved around the tumor site to ensure clean contact with the transducer and to remove potential artifacts from hair. Transducers were coupled to the mouse using clear ultrasound gel. 3D B-Mode, Oxyhemo, and Multi-Wavelength scans were conducted over the entire tumor volume prior to imaging. Oxyehmo imaging on Visualsonics systems, alternates between imaging at 750nm and 850nm and provides a live unmixed estimation of tissue oxygenation that can be adjusted based on total hemoglobin signal. Multi-wavelength scans included sequential imaging at 710, 780, 830, 890, 900, and 920nm at each position over the 3D volume. Wavelengths were chosen to allow spectral unmixing for IcG, LJA, HHb, and HbO_2_. Ground truth values for IcG in plasma were obtained from Landsman et al.^57^ 3D volume was selected based on tumor size under B-Mode imaging. Motor step-size is ~150μm. The imaging stage was heated to maintain the animal core body temperature at 37-39°C. Prior to injection, baseline signals were collected to allow calculation of contrast enhancement (CE) as the ratio of contrast agent signal at specific times after injection to the baseline signal. A 5mg/kg dose of either IcG or LJA (n=4), not exceeding 200μL, was injected intravenously via the tail vein. Mice were immediately imaged after injection (0hr or post-injection) as well as after 4, 8, and 24 hours. Unmixed signals were acquired from the Visualsonics VEVO LAB software with additional analysis performed on RAW data files using custom software written in MATLAB. Custom software separated the images files into 3D volumes allowing for ROIs to be drawn over the tumor region. Unmixed signals were averaged across the entire tumor volume for each mouse and normalized by the measured volume.

### 4.10. Biodistribution

Fluorescence intensity values obtained using a FOBI system from NPI Electronics (Tamm, Germany). Mice (n=4) received a 5mg/kg dose of liposomal IcG intravenously before being sacrificed 24 hours later. Mice were perfused with PBS to clear the blood and had their organs harvested. Fluorescence signal was background subtracted using control tissue and was normalized to the weight of the organ following imaging.

### 4.11. *In Vivo* Photothermal Therapy

Tumor-bearing mice were anesthetized and shaved around the tumor site to provide a clear laser-path. Mice were randomly placed in 4 groups: LJA, IcG, Laser Only, and Control. There were 5 mice per group for this stage of the study. Mice received a 5mg/kg dose of either IcG, LJA, or PBS (Laser Only/Control) intravenously. After 8 hours, the mice were again anesthetized and placed such that the tumor was in the path of the 852nm laser. Thermal camera was focused on the tumor site prior to 10 minutes of irradiation at 750mW/cm^2^. Temperature of the tumor site was collected for the entire 10 minutes. Temperature readings were used to calculate the more clinically relevant Cumulative Equivalent Minutes at 43°C (CEM43) using the equation



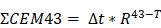



where ∆t is the time interval, T is the temperature, and R is a constant 0.25 if T < 43°C and 0.5 if T > 43°C. Following laser treatment, mice were monitored for up to two weeks. Tumor size and animal weight were monitored every other day to track tumor response. Mice were euthanized if tumors grew larger than IACUC standards (>500mm^3^) or if growth led to ulceration around the tumor or injection site.

## Supplementary Material

Supplementary figures.
