# Supplementary material for: Liposomal J-Aggregates of Indocyanine Green as a Multifunctional Contrast Agent for Photoacoustic Imaging and Photothermal Therapy
Source: Nanotheranostics. 2026 Feb 11;10:24–37. doi: 10.7150/ntno.123184 (PMC12923309; doi:10.7150/ntno.123184)
Supplement: Supplementary file 1 — Supplementary figures. [file ntnov10p0024s1.pdf]

Liposomal J-Aggregates of Indocyanine Green as a Multifunctional Contrast Agent for Photoacoustic Imaging and Photothermal Therapy

Noah Stern, Binita Shrestha, Susan Burrell, James Tunnell, Tyrone Porter*

Biomedical Engineering Department, University of Texas at Austin, Austin, TX, USA

Supplementary Figure 1:

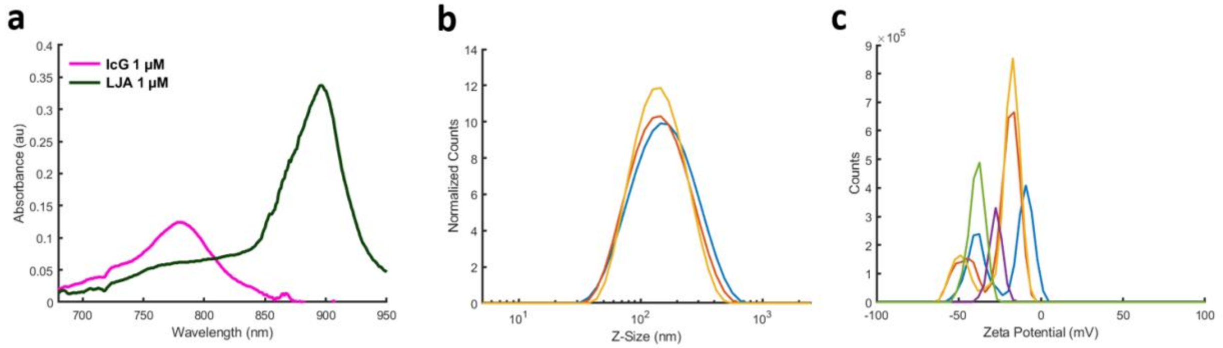

Supplementary Fig.1: (a) Absorbance, (b) Size, and (c) Zeta Potential of Liposomal J-Aggregate Particles. Individual plots in (b) and (c) represent data collected across trials individual batches of LJA. For example, each of the three lines shown in (b) depict the DLS reading from different batches of LJA synthesis. Individually the lines are averages of three technical replicates made per batch.

Supplementary Figure 2:

	808nm	*852nm*	*890nm*
**Beam Energy**	1 J	1 J	1 J
**Beam Radius**	4 mm	4mm	4mm
**g (anisotropy factor)**	0.79	0.79	0.79
**μ_a (epidermis)**	1.6 cm^{-1}	1.1 cm^{-1}	0.7 cm^{-1}
**μ_s' (epidermis)**	39.35 cm^{-1}	37.01 cm^{-1}	35.17 cm^{-1}
**μ_a (dermis)**	1.43 cm^{-1}	0.87 cm^{-1}	0.71 cm^{-1}
**μ_s' (dermis)**	20.11 cm^{-1}	22.75 cm^{-1}	23.51 cm^{-1}
**μ_a (fatty region)**	0.6 cm^{-1}	0.7 cm^{-1}	0.85 cm^{-1}
**μ_s' (fatty region)**	10.12 cm^{-1}	9.57 cm^{-1}	9.14 cm^{-1}
**μ_a (tumor region LJA)**	6.56 cm^{-1}	11.56 cm^{-1}	22.12 cm^{-1}
**μ_s' (tumor region LJA)**	21.29 cm^{-1}	21.61 cm^{-1}	21.93 cm^{-1}
**μ_a (tumor region blank)**	2.12 cm^{-1}	1.98 cm^{-1}	1.55 cm^{-1}
**μ_s' (tumor region blank)**	21.11 cm^{-1}	21.43 cm^{-1}	21.75 cm^{-1}

Supplementary Fig. 2: Values used for MCML and CONV programs.

Supplementary Figure 3:

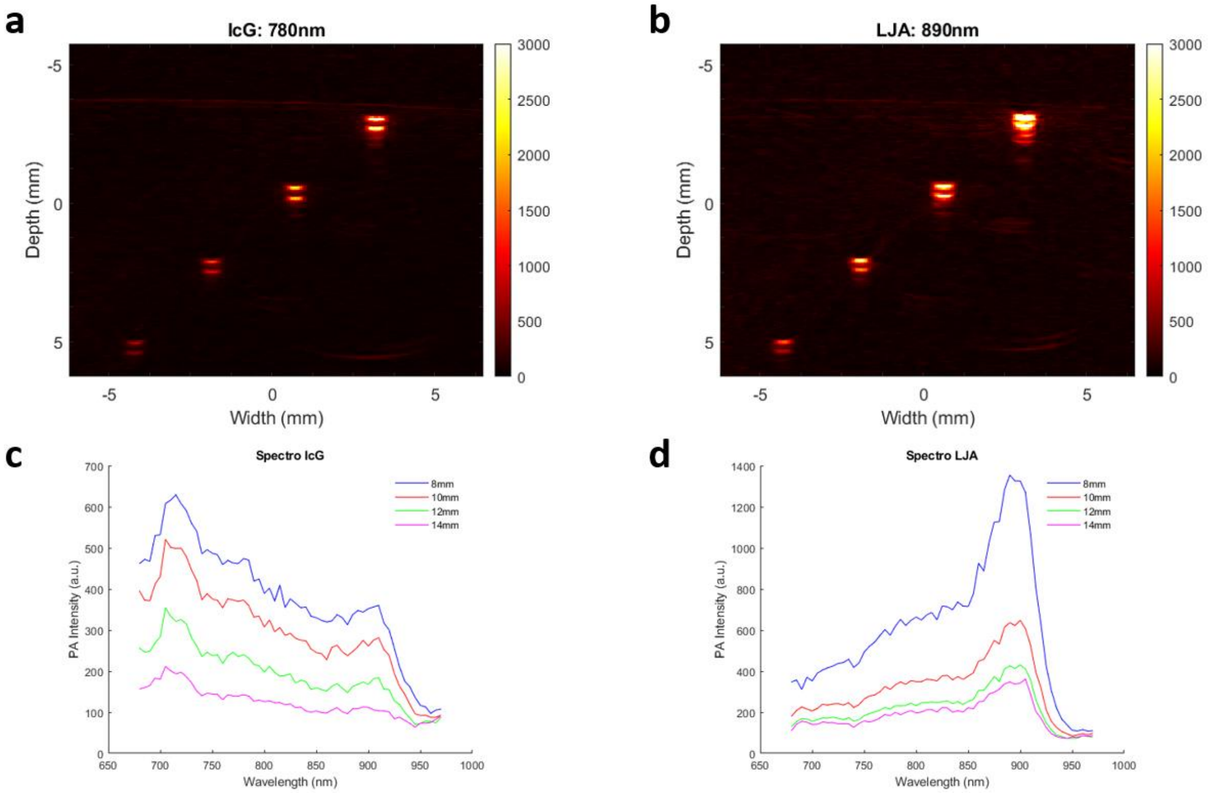

Supplementary Fig. 3: PA Images of LJA and IcG at Peak Wavelengths Obscured by Tissue Mimicking Phantoms. For IcG images are at 780nm (a) and for LJA images are at 890nm(b). Spectral profiles, values obtained every 5nm, are included for both sets of IcG (c) and LJA (d). At high concentration IcG peaks closer to 710 due to dimerization of IcG monomers.

<u>**Supplementary Figure 4:**</u>

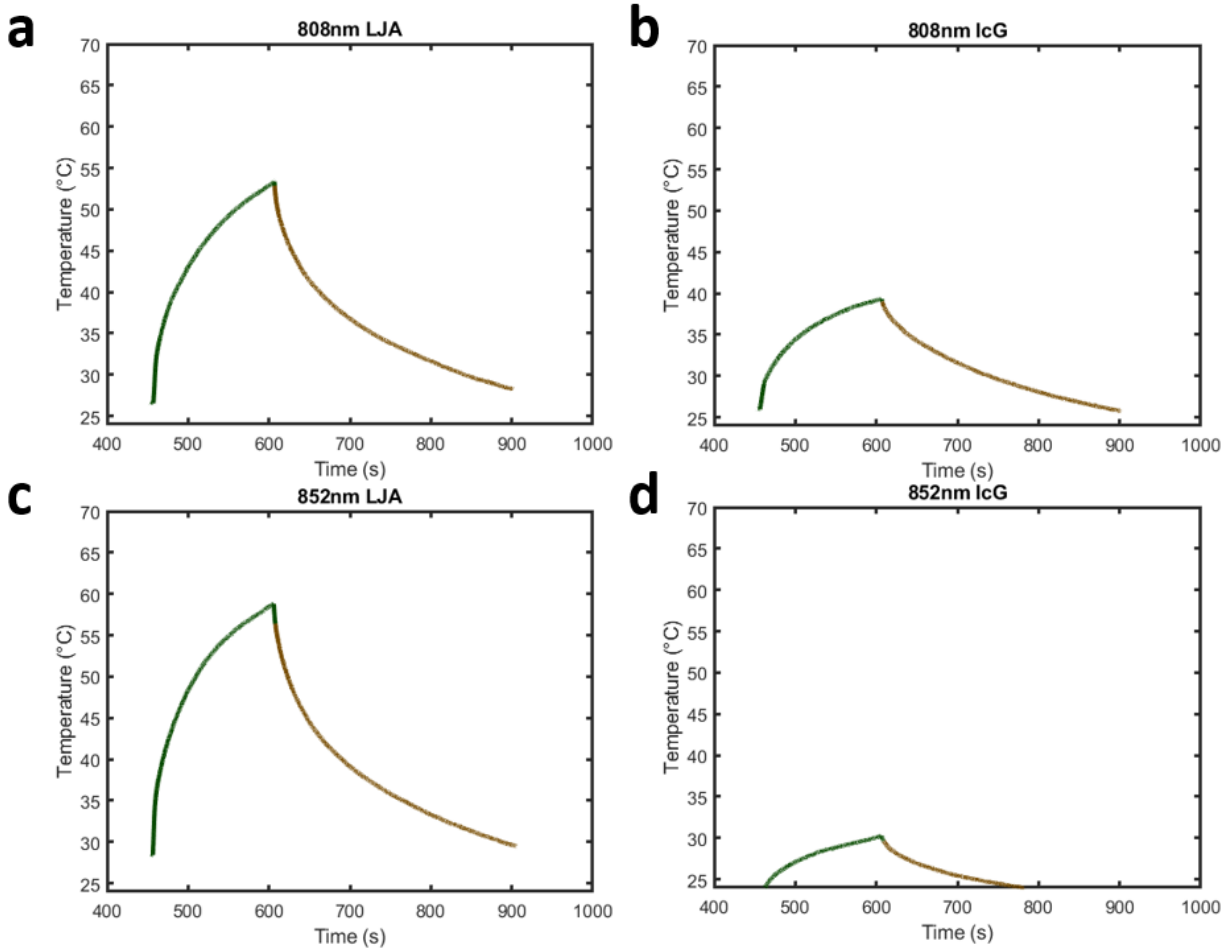

Supplementary Fig. 4: Heating and Cooling Curves for IcG and LJA PTT at 808nm and 852nm

Supplementary Figure 5:

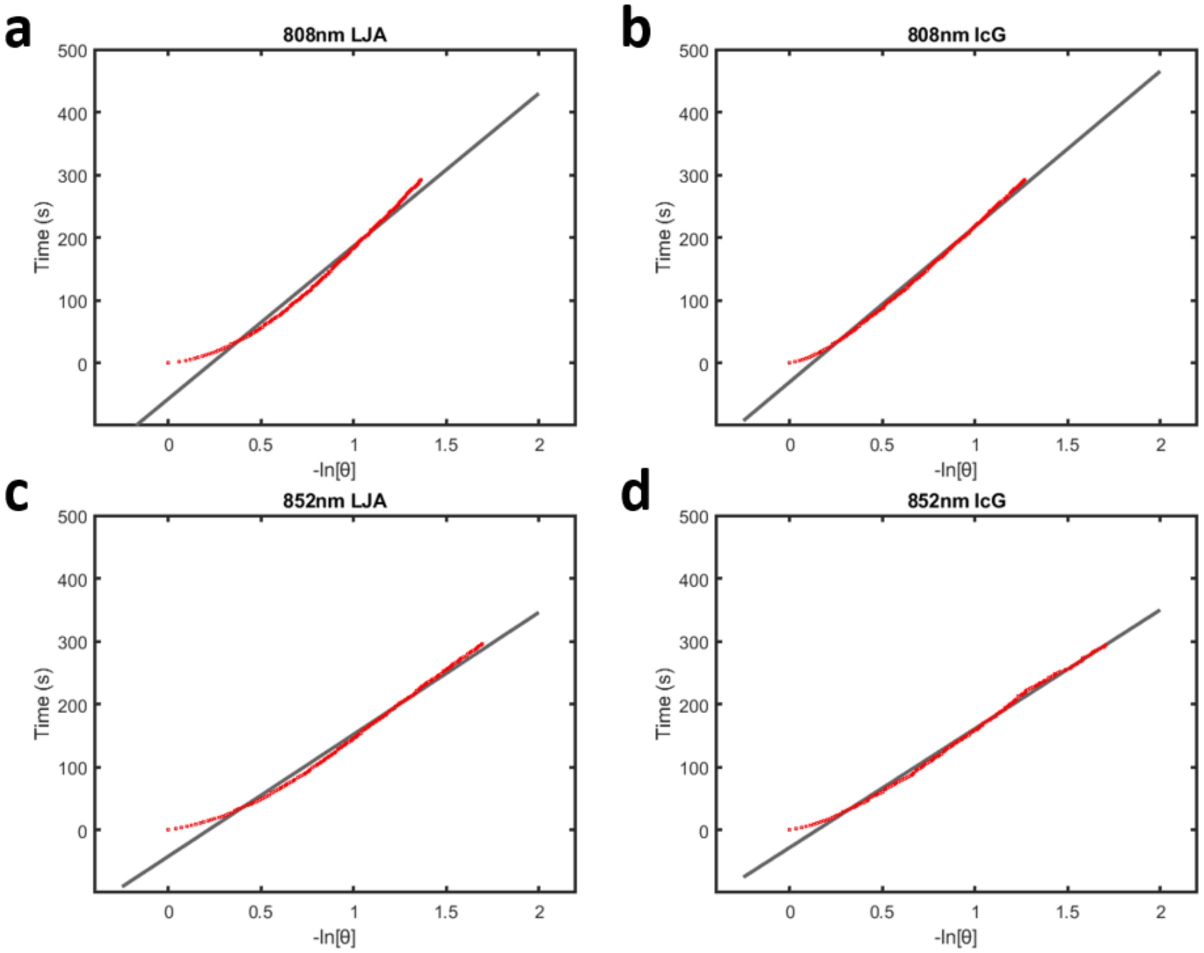

Supplementary Fig. 5: Linearized Cooling Curves for IcG and LJA PTT at 808nm and 852nm

Supplementary Figure 6:

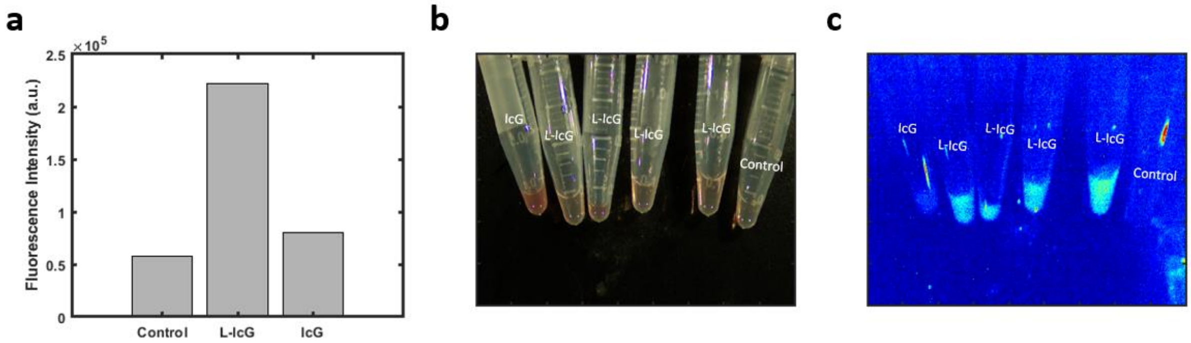

Supplementary Fig. 6: Liposome and Free IcG content in blood 24 hours after injection. (a) Average fluorescence intensity of control, L-IcG, and IcG in serum normalized to volume of serum. (b) Brightfield image of collected serum. (c) Fluorescence intensity image of control, L-IcG, and IcG serum.

Supplementary Figure 7:

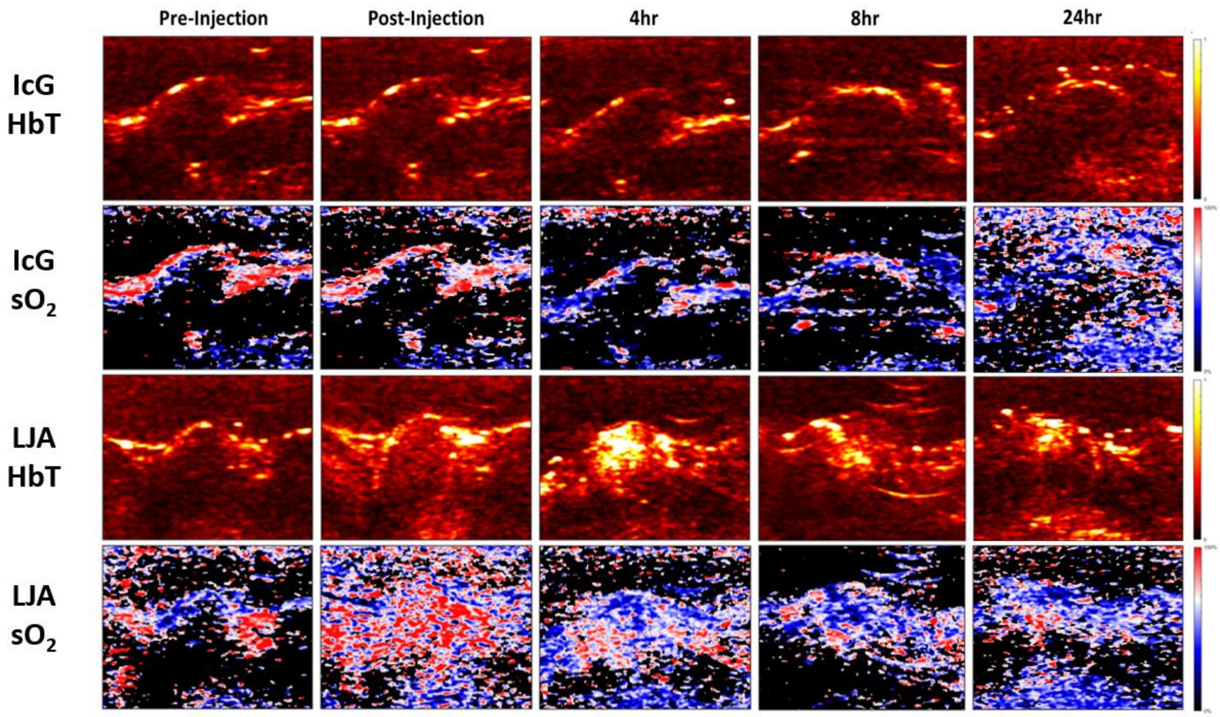

Supplementary Fig. 7: HbT and sO_2 maps for IcG and LJA at each imaging time point.

Supplementary Figure 8:

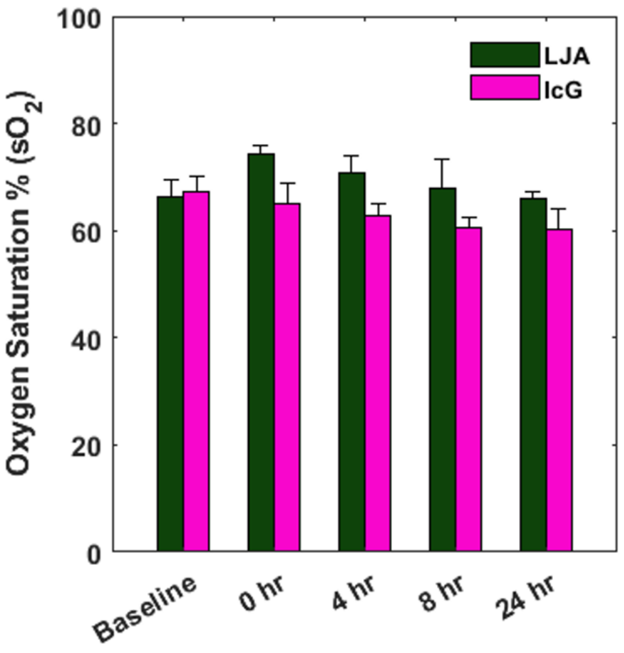

Supplementary Fig. 8: Measured/estimated sO_2 values at each time point for both groups.

Supplementary Figure 9:

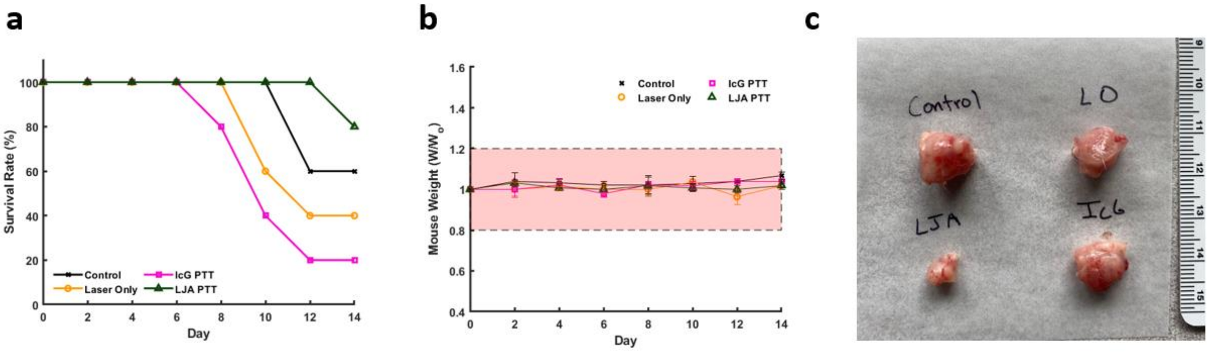

Supplementary Fig. 9: (a) Survival rate, (b) weight, and (c) images of resected tumors after study conclusion.

Supplementary Figure 10:

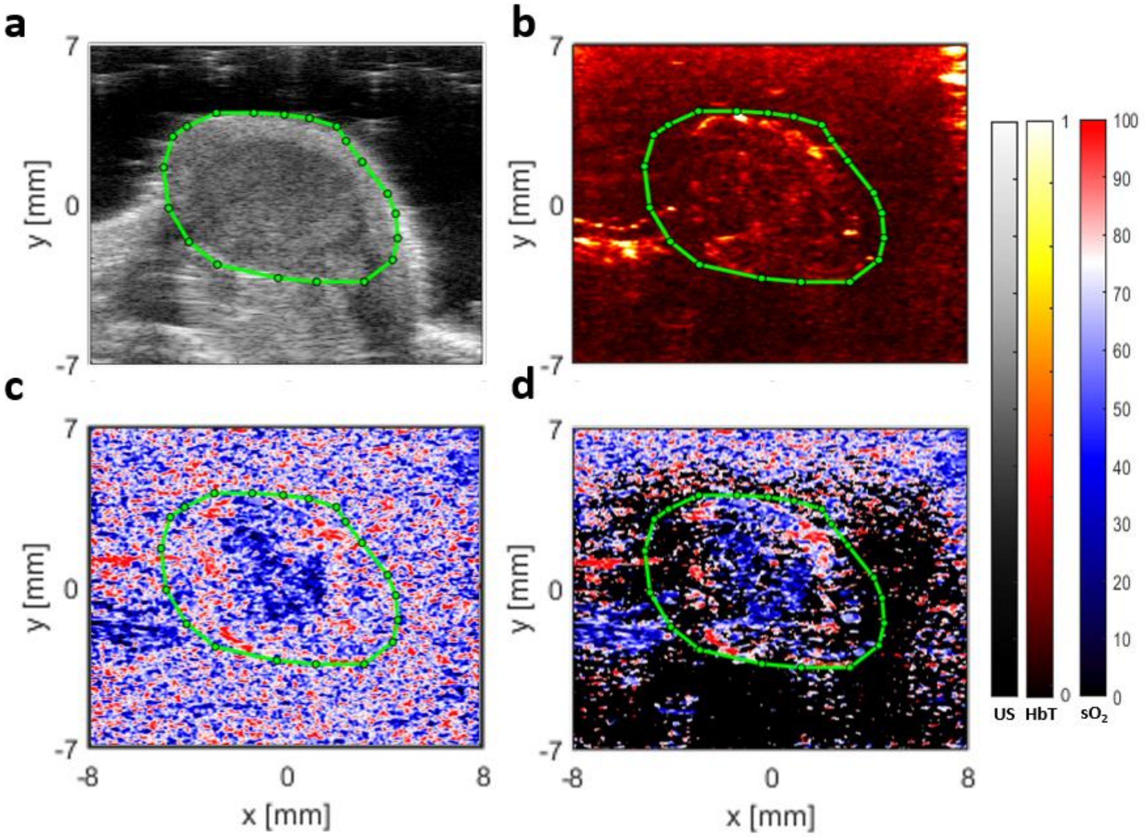

Supplementary Fig. 10: Example workflow including (a) ultrasound, (b) total hemoglobin, (c) sO_2, and (d) thresholded sO_2 images.

Supplementary Figure 11:

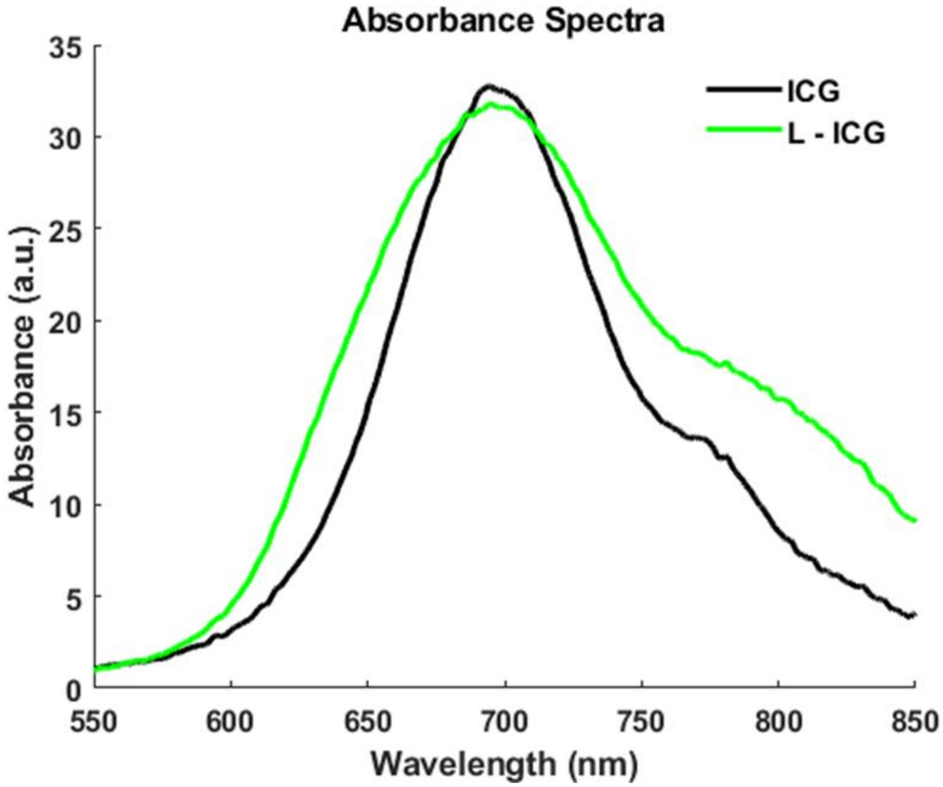

Supplementary Fig. 11: Spectrophotometer readings of IcG and L-IcG. Absorption spectra of equal concentrations (in-terms of total ICG content) between unencapsulated and liposome-encapsulated ICG is mostly unchanged. Some slight broadening of the spectra occurs due to interactions with the lipid bilayer. For L-ICG, some of the ICG molecules likely exist in the monomeric form (peak: ~780nm) within the bilayer as opposed to the dimer form (peak: ~710nm) resulting in the slight absorption increase at longer wavelengths. As such it can be inferred that J-aggregation, not the liposome shell itself leads to the significant redshift seen for LJA.

Supplementary Figure 12:

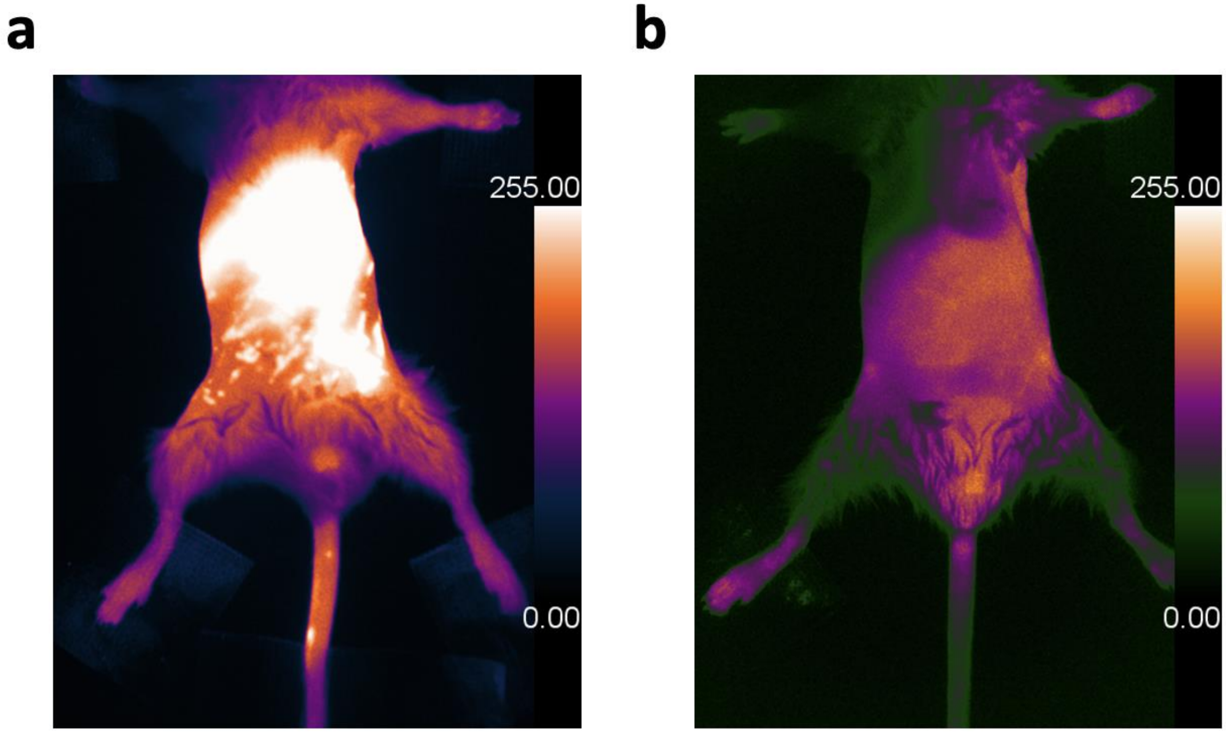

Supplementary Fig. 12: Fluorescence intensity of mouse abdomen 60 seconds after injection of (a) IcG and (b) LJA. Interestingly, unlike some other J-aggregates of cyanine dyes, J-aggregates of IcG are much less fluorescent than monomeric IcG.
